# The association between adherence to national antibiotic guidelines and mortality, readmission and length of stay in hospital inpatients: results from a Norwegian multicentre, observational cohort study

**DOI:** 10.1186/s13756-019-0515-5

**Published:** 2019-04-15

**Authors:** Jannicke Slettli Wathne, Stig Harthug, Lars Kåre Selland Kleppe, Hege Salvesen Blix, Roy M. Nilsen, Esmita Charani, Ingrid Smith

**Affiliations:** 1 Department of Clinical Science, University of Bergen, Jonas Lies vei 87, 5021 Bergen, Norway; 2 Norwegian Advisory Unit for Antibiotic Use in Hospitals, Department of Research and Development, Haukeland University Hospital, Jonas Lies vei 65, 5021 Bergen, Norway; 3 Department of Quality and Development, Hospital Pharmacies Enterprise in Western Norway, Møllendalsbakken 9, 5021 Bergen, Norway; 4 Department of Infectious Diseases and Unit for Infection Prevention and Control, Department of Research and Education, Stavanger University Hospital, Armauer Hansens vei 20, 4011 Stavanger, Norway; 5 Department of Drug Statistics, Norwegian Institute of Public Health, Marcus Thranes gate 6, 0473 Oslo, Norway; 6 Western Norway University of Applied Sciences, Inndalsveien 28, 5063 Bergen, Norway; 7 NHIR Health Protection Research Unit in Healthcare Associated Infections and Antimicrobial Resistance, Imperial College London, Hammersmith Hospital Campus, Du Cane Road, W12 0NN, London, UK; 8 Innovation, Access and Use, Department of Essential Medicines and Health Products, World Health Organization (WHO), Avenue Appia 20, 1211, 27 Geneva, Switzerland

**Keywords:** Antimicrobial stewardship, Antibiotic stewardship, Antibiotic guidelines, Adherence, Patient outcome, Mortality, Readmission, Length of stay

## Abstract

**Background:**

Clinical antibiotic prescribing guidelines are essential in defining responsible use in the local context. Our objective was to investigate the association between adherence to national antibiotic prescribing guidelines and patient outcomes across a wide range of infectious diseases in hospital inpatients.

**Methods:**

Over five months in 2014, inpatients receiving antibiotics under the care of pulmonary medicine, infectious diseases and gastroenterology specialties across three university hospitals in Western Norway were included in this observational cohort study. Patient and antibiotic prescribing data gathered from electronic medical records included indication for antibiotics, microbiology test results, discharge diagnoses, length of stay (LOS), comorbidity, estimated glomerular filtration rate (eGFR) on admission and patient outcomes (primary: 30-day mortality; secondary: in-hospital mortality, 30-day readmission and LOS). Antibiotic prescriptions were classified as adherent or non-adherent to national guidelines according to documented indication for treatment. Patient outcomes were analysed according to status for adherence to guidelines using multivariate logistic, linear and competing risk regression analysis with adjustments made for comorbidity, age, sex, indication for treatment, seasonality and whether the patient was admitted from an institution or not.

**Results:**

In total, 1756 patients were included in the study. 30-day-mortality and in-hospital mortality were lower (OR = 0.48, *p* = 0.003 and OR = 0.46, *p* = 0.001) in the guideline adherent group, compared to the non-adherent group. Adherence to guideline did not affect 30-day readmission. In linear regression analysis there was a trend towards shorter LOS when LOS was analysed for patients discharged alive (predicted mean difference − 0.47, 95% CI (− 1.02, 0.07), *p* = 0.081). In competing risk analysis of LOS, the adherent group had a subdistribution hazard ratio (SHR) of 1.17 95% CI (1.02, 1.34), *p* = 0.025 for discharge compared to the non-adherent group.

**Conclusions:**

Adhering to antibiotic guidelines when treating infections in hospital inpatients was associated with favourable patient outcomes in terms of mortality and LOS.

**Electronic supplementary material:**

The online version of this article (10.1186/s13756-019-0515-5) contains supplementary material, which is available to authorized users.

## Background

Antibiotics constitute an important class of medicines, where the use of a substance has implications beyond the patient being treated. Antimicrobial stewardship is a systematic way to improve antibiotic use in hospitals and has most recently been defined as “a coherent set of actions which promote using antimicrobials responsibly” [1]. Clinical guidelines for antibiotic use are essential in defining responsible use in the local context and are one of the core elements of stewardship programmes [2]. Studying the association between antibiotic use and patient outcomes is of great importance and can imply whether guideline-adherent prescribing practice is safe and secures equal – or better patient outcome. Most studies in this field are performed within lower respiratory tract infections and many are prone to confounding by indication, because patients with less severe illness are more likely to have received the more narrow-spectrum, guideline adherent therapy [3].

Norway has low, but steadily increasing antibiotic resistance rates [4]. Seven months prior to this study, new national guidelines for antibiotic use in hospitals were published [5]. We aimed to investigate if appropriate prescribing practices for hospitalised patients with a broad spectrum of infectious diseases were associated with patient outcomes when adjusted for major confounding factors.

## Methods

### Study design and setting

We performed an observational study in the cohort of patients from a previously published cluster randomized controlled intervention study, which was performed at three emergency care and teaching hospitals in Western Norway [6]. Hospital A and B are tertiary care hospitals with 1100 and 600 beds, respectively. Hospital C is a secondary care hospital with 160 beds. Hospital A is in addition referral hospital for hospitals B and C. Three medical wards from hospital A and B (infectious diseases, pulmonary medicine and gastroenterology) and two medical wards from hospital C (infectious diseases/general medicine and pulmonary/cardiac medicine) were included in the study. All hospitals were committed to be using the national guideline for antibiotic use for hospital inpatients [5].

### Data collection

Adult patients (over 18 years old) were included in the study if they received antibiotics for a suspected or confirmed infection during admission, were discharged from a study ward between the 10th of February and the 11th of July 2014 and had a hospital stay of > 24 h and ≤ 21 days. Minimum length of hospital stay was defined to assure that included patients were seen by study ward physicians and maximum length to make manual data collection throughout the hospital stay feasible. Patients who either only received antibiotic prophylaxis, had orthopaedic prosthesis infections or had an indication for treatment not covered by the national guidelines, were not included. For patients readmitted during the study period, only the first stay was included in analysis. Where data regarding outcome was not possible to retrieve (e.g. tourists), or comorbidity data was unavailable, the patient was excluded. Data were collected manually from electronic medical records, including admission notes, medical charts, physician’s notes, discharge letters and laboratory results. Data included patient demographics, indication for antibiotic treatment, antibiotic use, microbiology test results, estimated glomerular filtration rate (eGFR) on admission, length of stay (LOS), 30-day readmission, in-hospital and 30-day mortality, comorbidity and admittance from- or discharge to institution. Mortality data was continuously updated within the electronic medical record, using data from the Norwegian National Registry [7] Supplementary data on main diagnosis at discharge and comorbidity was retrieved by extraction from electronic medical records. Readmissions were only captured if patients were readmitted to the same hospital as the patient was discharged from.

### Definitions

All substances comprising the ATC-group “Antibacterials for systemic use” (J01), metronidazole tablets (P01AB01) and vancomycin tablets (A07AA09) were included in the definition of antibiotics for this study [8].

### Outcome measures

Primary outcome measure was 30-day mortality, defined as all-cause mortality during hospital stay or within 30 days of discharge from hospital.

Secondary outcome measures were

- In-hospital mortality, defined as all-cause in-hospital mortality during study admission.
- 30-day readmission, defined as all-cause acute readmission to the same study hospital as the patient was discharged from, within 30 days of discharge, for patients discharged alive and not transferred to another hospital.
- Length of stay, defined as number of days from admission to discharge for the entire hospital stay for patients discharged alive, except for time spent at a hospital rehabilitation centre after discharge from a study ward. LOS was also analysed for all patients, with in-hospital mortality as competing risk.

### Study variable

Adherence to national antibiotic guidelines refers to the choice of active substance(s) for the initial indication for treatment. Dosing of the substance(s) was not considered. Adherence was assessed by using syntax in SPSS, combining the variable for indication for treatment with the variable for prescribed treatment. Only the first-choice empirical regimens were regarded adherent. For patients with antibiotic allergies or kidney failure where chosen treatment was an alternative guideline regimen (not first choice), manual adjustment of the adherence variable was performed consistently throughout the study population. CRB-65-score^1^ and the severity of pneumonia were usually not explicitly stated in the patient notes. Less severe and severe community acquired pneumonia were therefore assessed together, meaning that first line treatments for both conditions were considered adherent. Some patients had more than one working diagnosis on initiation of therapy. An ID-physician (BS) reviewed the diagnoses and decided indication for treatment for these patients, expecting initial therapy to be based on the most severe working diagnosis. Infections described as “suspected pneumonia” or “unspecified lower respiratory tract infection” on admission were assessed for adherence as community- or hospital acquired pneumonia. A working diagnosis as “suspected urinary tract infection” (UTI) was assessed as adherent if treatment was according to guideline treatment for either pyelonephritis or cystitis. For the indication “suspected pneumonia/UTI”, treatment according to guideline recommendation for either diagnosis were considered adherent.

### Adjustment variables

Indication for treatment was the indication for first treatment with antibiotics and was always an infection. Physicians’ notes were used to identify indication for treatment and indication was not further assessed for validity. Indications were grouped into six main categories (Table 1). Indications which did not fit into the main categories were included in a seventh category of “Other infections”. Empirical antibiotic treatment was specific for each indication and varied within each group.

**Table 1:** Grouping of indications for treatment

	Indication for treatment
Lower respiratory tract infections (LRTI)	Community acquired pneumonia (normal and severe), healthcare associated pneumonia (normal and severe), unspecified lower respiratory tract infections, unknown – suspected pneumonia, aspiration pneumonia, atypical pneumonia, lung abscess, empyema.
Acute exacerbations of chronic obstructive pulmonary disease (COPD with LRTI)	Patients with COPD, presenting with LRTI (community and healthcare associated)
Sepsis	Focus area; lower respiratory tract, urinary tract, unknown focus, soft tissue, abdomen and catheter.
Skin and soft tissue infections (SSTI)	Erysipelas, cellulitis, abscess, other skin and soft tissue infections, mastitis, necrotising soft tissue infections, postoperative wound infection.
Gastrointestinal tract infections (GI-infections)	Helicobacter pylori-infection, gastroenteritis, peritonitis, cholecystitis/cholangitis, *Clostridium difficile* (C.Diff).
Urinary tract infections (UTI)	UTI – unspecified, pyelonephritis, lower UTI/cystitis, unknown-suspected UTI, catheter associated UTI.
Other infections	Suspected both pneumonia and UTI, meningitis, neutropenic fever, osteomyelitis, tonsillitis, arthritis, endocarditis, sinusitis/otitis, and infected intravascular catheters

Indications within each group are given in decreasing order of frequency

Comorbidity was defined using the Charlson Comorbidity Index (CCI) [9, 10]. For each patient, up to eight diagnoses were extracted from the hospital electronic medical record at discharge. All extracted diagnoses were included in the calculation of CCI, using Stata syntax [11]. Estimated glomerular filtration rate on admission was originally planned as an adjustment variable, but as renal disease is included in CCI, this was discarded from analysis. Age was coded in age groups, starting with patients up to and including the age of 45 and thereafter given in groups of 20 years to the last group of above 85 years. Admission from an institution was defined as patients admitted at an institution with 24/7 care, e.g. another hospital or nursing home, within 48 h of admission. Adjustment for seasonality was performed by using the week of admission as adjustment variable.

### Statistics/analysis

To analyse differences in patient characteristics between the groups with adherent and non-adherent treatment, we used chi-square test and two-sample t-test for categorical and continuous data, respectively. Univariate and multivariate logistic and linear regression were used to study the association between guideline adherent prescribing practice and patient outcome. Indication for treatment, comorbidity (CCI), age group, admittance from institution, sex and seasonality (week of admission) were evaluated as adjustment variables. Variables that in univariate regression analysis of 30-day mortality had a *p*-value of less than 0.2 (all evaluated variables) were included in multivariate analyses for all studied outcomes. In addition, we used robust variance estimation of regression coefficients to account for clustered observations on the same hospital ward.

Two sensitivity analyses were performed for 30-day mortality. In the first, grouping of indication for treatment was replaced by grouped discharge diagnoses as adjustment variable to evaluate whether estimates of association would change if diagnoses had changed from admission to discharge. In the second sensitivity analysis, grouping of indication for treatment was replaced by individual indications as an adjustment variable to evaluate whether the grouping of indications could influence the results.

As the linear regression models of LOS did not account for in-hospital mortality, we also performed a sensitivity analyses for this outcome by fitting a Fine-Gray model with in-hospital mortality as competing risk. In this analysis, we report associations as the subdistribution hazard ratio (SHR) with 95% confidence intervals, which denotes the magnitude of the relative difference in the subdistribution hazard function between adherent and non-adherent groups [12].

A *p*-value of < 0.05 was considered statistically significant for all analyses. Statistical analysis was performed using Stata SE version 15 (Stata Statistical Software, College Station, TX, USA).

## Results

During the study period, 1783 patients were eligible for inclusion. We were not able to retrieve comorbidity data for 22 patients. For 5 patients who were tourists, outcome data was unavailable. In final analyses, 1756 patients were therefore included.

There was a significant difference between the adherent and non-adherent group with regards to the groups of indication for treatment, with a higher percentage of LRTI’s in the adherent group and more patients with GI-infections, UTIs and “other” infections in the non-adherent group (Table 2). The non-adherent group also had a higher proportion of patients admitted from an institution.

**Table 2:** Patient characteristics and outcome by adherence or non-adherence to guidelines

	Non-adherence (*N* = 667)	Adherence (*N* = 1089)	*P*-value
Patient characteristics			
Indication for treatment			
LRTI	161 (24.1)	372 (34.2)	**< 0.001**
COPD with LRTI	124 (18.6)	230 (21.1)	
Sepsis	111 (16.6)	180 (16.5)	
SSTI	72 (10.8)	115 (10.6)	
GI-infection	44 (6.6)	34 (3.1)	
UTI	80 (12.0)	99 (9.1)	
Other infections	75 (11.2)	59 (5.4)	
Charlson Comorbidity Index			
CCI = 0	240 (36.0)	432 (39.7)	0.083
CCI = 1	212 (31.8)	373 (34.3)	
CCI = 2	119 (17.8)	141 (13.0)	
CCI = 3	45 (6.8)	65 (6.0)	
CCI = 4	24 (3.6)	33 (3.0)	
CCI > 4	27 (4.1)	45 (4.1)	
Age, mean (std.dev.)	67.3 (18.2)	67.4 (19.1)	0.885
Age			
<=45	92 (13.8)	161 (14.8)	0.680
46–65	156 (23.4)	240 (22.0)	
66–85	320 (48.0)	508 (46.7)	
>85	99 (14.8)	180 (16.5)	
Admitted from institution	120 (18.0)	135 (12.4)	**0.001**
Discharged to institution	182 (29.0)	258 (24.4)	0.090
Sex			
Male	352 (52.8)	565 (51.9)	0.717
Female	315 (47.2)	524 (48.1)	
Outcome			
In-hospital mortality	38 (5.7)	32 (2.9)	**0.004**
30-day mortality	75 (11.2)	67 (6.2)	**< 0.001**
30-day readmission (*n* = 623/1047)	140 (22.5)	206 (19.7)	0.173
LOS^a^, mean (std.dev.) (*n* = 623/1047)	7.3 (4.4)	6.7 (4.1)	**0.004**

^a^*LOS* = Length of stay. All analysis was performed using chi-square tests, except mean age and LOS which were analysed using two-sample t-test. *P*-values in boldface are statistically significant (<0.05)

Thirty-day mortality and in-hospital mortality was significantly lower in patients receiving guideline adherent treatment, with an odds ratio (OR) of 0.48, with *p* = 0.003 for 30-day mortality and OR = 0.46, with *p* = 0.001 for in-hospital mortality (Table 3).

**Table 3:** Adjusted analysis of the association between guideline adherence, in-hospital and 30-day mortality

	All patients	In-hospital mortality			30-day mortality		
	(*N* = 1756)	(n_1_ = 70)	(*N* = 1756)	P	(n_2_ = 142)	(*N* = 1756)	P
	n (%)	n (%)	OR (95% CI)		n (%)	OR (95% CI)	
Adherence to guideline							
No	667 (38.0)	38 (5.7)	1.00		75 (11.2)	1.00	
Yes	1089 (62.0)	32 (2.9)	0.46 (0.29, 0.74)	0.001	67 (6.2)	0.48 (0.29, 0.78)	0.003
Indication for antibiotic treatment							
LRTI	533 (30.4)	35 (6.6)	1.00		69 (13.0)	1.00	
COPD with LRTI	354 (20.2)	11 (3.1)	0.44 (0.22, 0.86)	0.017	22 (6.2)	0.45 (0.35, 0.59)	< 0.001
Sepsis	291 (16.6)	14 (4.8)	0.69 (0.41, 1.15)	0.153	24 (8.3)	0.59 (0.36, 0.97)	0.038
SSTI	187 (10.7)	1 (0.5)	0.12 (0.02, 0.66)	0.015	3 (1.6)	0.17 (0.03, 1.09)	0.061
GI-infection	78 (4.4)	3 (3.9)	0.75 (0.10, 5.72)	0.782	6 (7.7)	0.78 (0.22, 2.80)	0.708
UTI	179 (10.2)	2 (1.1)	0.12 (0.27, 0.55)	0.006	11 (6.2)	0.35 (0.19, 0.63)	0.001
Other infections	134 (7.6)	4 (3.0)	0.35 (0.17, 0.72)	0.004	7 (5.2)	0.29 (0.19, 0.46)	< 0.001
Charlson Comorbidity Index							
CCI = 0	672 (38.3)	9 (1.3)	1.00		20 (3.0)	1.00	
CCI = 1	585 (33.3)	19 (3.3)	1.60 (0.52, 4.86)	0.411	36 (6.2)	1.40 (0.95, 2.04)	0.088
CCI = 2	260 (14.8)	16 (6.2)	2.66 (0.72, 9.83)	0.143	31 (11.9)	2.67 (1.45, 4.90)	**0.002**
CCI = 3	110 (6.3)	6 (5.5)	2.27 (0.60, 8.60)	0.228	16 (14.6)	3.18 (1.75, 5.78)	**< 0.001**
CCI = 4	57 (3.3)	8 (14.0)	6.39 (1.64, 24.93)	**0.008**	14 (24.6)	6.79 (3.31, 13.95)	**< 0.001**
CCI > 4	72 (4.1)	12 (16.7)	8.50 (3.80, 19.04)	**< 0.001**	25 (34.7)	12.04 (8.02, 18.08)	**< 0.001**
Age							
< =45	253 (14.4)	1 (0.4)	1.00		2 (0.8)	1.00	
46–65	396 (22.6)	7 (1.8)	2.35 (0.35, 15.70)	0.376	14 (3.5)	2.40 (0.53, 10.87)	0.257
66–85	828 (47.2)	39 (4.7)	5.42 (0.82, 35.67)	0.079	80 (9.7)	5.61 (1.51, 20.85)	**0.010**
> 85	279 (15.9)	23 (8.2)	10.13 (0.99, 103.78)	0.051	46 (16.5)	9.81 (1.91, 50.36)	**0.006**
Admitted from institution							
No	1501 (85.5)	46 (3.1)	1.00		88 (5.9)	1.00	
Yes	255 (14.5)	24 (9.4)	2.53 (1.45, 4.43)	**0.001**	54 (21.2)	3.74 (2.69, 5.20)	**< 0.001**
Sex							
Male	917 (52.2)	49 (5.3)	1.00		88 (9.6)	1.00	
Female	839 (47.8)	21 (2.5)	0.42 (0.28, 0.61)	**< 0.001**	54 (6.4)	0.59 (0.39, 0.90)	**0.015**
Week of admission^a^			0.96 (0.93, 0.99)	**0.005**		0.96 (0.93, 0.997)	**0.031**

^a^Adjustment for seasonality was performed by using the week of admission as adjustment variable

In-hospital – and 30-day mortality was analysed using multivariate, logistic regression analysis with adjustment for clustering at individual sites. All variables are included in adjusted analysis. *P*-values in boldface are statistically significant (<0.05)

During admission, 70 patients died and 16 patients were discharged to another hospital, so in analysis of 30-day readmission and LOS, 1670 patients were included (Table 4). There was no evidence of any differences in 30-day readmission between patients receiving guideline adherent treatment or not. Comorbidity (CCI) and seasonality (the week of admission) were the only variables significantly associated with 30-day readmission. In the linear regression analysis of LOS, there was a trend towards shorter LOS when guideline adherent treatment was prescribed at treatment onset (− 0.47 days, *p* = 0.087) (Table 4). This result was supported by the competing risk analyses of LOS in which the adherent group was associated with a 17% increase in the rate of discharge, compared with the non-adherent group (Additional file 1: Table S1; SHR 1.17, 95% CI (1.02, 1.34), *p* = 0.025).

**Table 4:** Adjusted analysis of the association between guideline adherence, 30-day readmission and length of stay

	All patients	30 day readmission			Length of stay		
	(*N* = 1670)	(*N* = 346)	(*N* = 1670)	P	(*N* = 1670)	(*N* = 1670)	P
	n (%)	n (%)	OR (95%CI)		Mean (S.D)	Coeff. (95% C.I.)	
Adherence to guideline							
No	623 (37.3)	140 (22.5)	1.00		7.3 (4.4)		
Yes	1047 (62.7)	206 (19.7)	0.87 (0.67, 1.14)	0.321	6.7 (4.1)	−0.47 (−1.02, 0.07)	0.081
Indication for antibiotic treatment							
LRTI	492 (29.5)	100 (20.3)	1.00		7.0 (4.3)		
COPD with LRTI	341 (20.4)	88 (25.8)	1.17 (0.80, 1.73)	0.421	6.6 (3.8)	−0.79 (−1.65, 0.08)	0.069
Sepsis	275 (16.5)	46 (16.7)	0.81 (0.54, 1.21)	0.303	7.0 (3.9)	0.22 (−0.73, 1.18)	0.605
SSTI	184 (11.0)	29 (15.8)	0.89 (0.62, 1.28)	0.522	6.2 (4.1)	−0.17 (−1.44, 1.10)	0.761
GI-infection	75 (4.5)	18 (24.0)	1.26 (0.64, 2.51)	0.503	7.3 (4.2)	0.53 (− 0.75, 1.81)	0.363
UTI	176 (10.5)	43 (24.4)	1.30 (0.85, 2.01)	0.229	7.1 (4.4)	0.10 (−0.74, 0.95)	0.781
Other infections	127 (7.6)	22 (17.3)	0.78 (0.51, 1.18)	0.240	7.5 (5.1)	0.53 (−0.83, 1.89)	0.386
Charlson Comorbidity Index							
CCI = 0	656 (39.3)	97 (14.8)	1.00		6.3 (3.9)		
CCI = 1	562 (33.7)	117 (20.8)	1.35 (1.03, 1.76)	**0.029**	6.9 (4.0)	0.60 (−0.32, 1.53)	0.168
CCI = 2	241 (14.4)	73 (30.3)	2.26 (1.46, 3.52)	**< 0.001**	7.3 (4.2)	0.87 (0.06, 1.68)	**0.039**
CCI = 3	103 (6.2)	27 (26.2)	1.77 (1.12, 2.82)	**0.015**	7.7 (5.0)	1.30 (−0.79, 3.38)	0.185
CCI = 4	49 (2.9)	16 (32.7)	2.55 (1.73, 3.76)	**< 0.001**	9.1 (5.4)	2.64 (− 0.42, 5.70)	0.081
CCI > 4	59 (3.5)	16 (27.1)	1.88 (0.89, 3.95)	0.098	9.1 (5.2)	2.42 (1.15, 3.69)	**0.003**
Age							
< =45	250 (15.0)	37 (14.8)	1.00		5.7 (4.1)		
46–65	387 (23.2)	75 (19.4)	1.07 (0.71, 1.62)	0.743	6.6 (4.0)	0.74 (0.45, 1.03)	**0.001**
66–85	779 (46.7)	175 (22.5)	1.15 (0.70, 1.90)	0.576	7.3 (4.3)	1.20 (0.58, 1.83)	**0.002**
> 85	254 (15.2)	59 (23.2)	1.24 (0.62, 2.49)	0.549	7.2 (4.3)	1.00 (−0.19, 2.18)	0.087
Sex							
Male	856 (51.3)	182 (21.3)	1.00		6.9 (4.3)		
Female	814 (48.7)	164 (20.2)	0.94 (0.81, 1.09)	0.411	6.9 (4.1)	0.02 (−0.64, 0.69)	0.942
Admitted from institution							
No	1441 (86.3)	301 (20.9)	1.00		6.8 (4.2)		
Yes	229 (13.7)	45 (19.7)	0.85 (0.62, 1.16)	0.307	7.2 (4.5)	0.02 (−0.53, 0.57)	0.938
Week of admission^a^			0.98 (0.96, 1.00)	**0.040**		−0.05 (− 0.09, − 0.002)	**0.044**

^a^Adjustment for seasonality was performed by using the week of admission as adjustment variable

All variables are included in adjusted analysis. 30-day readmission and length of stay was analysed using multivariate logistic- and linear regression, respectively with adjustment for clustering at individual sites. *P*-values in boldface are statistically significant (<0.05)

### Other analysis

We performed two sensitivity analyses for 30-day mortality. In the first analysis, grouped indications for treatment were substituted with grouped discharge diagnoses, which could be infections or non-infections. The association between adherent treatment and mortality now had an OR = 0.51, 95% CI (0.33, 0.80) with *p* = 0.003 (not shown in tables). In the second analysis, grouped indications were substituted with the individual indications in the regression model. This changed the estimated OR from 0.48 to 0.54, 95% CI (0.30, 0.99), *p* = 0.045 (not shown in tables). For the last analysis, model fit was poor for indications with few patients and no observed mortality. Only 1591 patients were kept in the model for this analysis.

## Discussion

The main findings of this study are that adherence to antibiotic guidelines at initiation of antibiotic therapy is associated with lower in-hospital- and 30-day mortality and shorter LOS. Adherence to guidelines was not significantly associated with 30-day readmission.

Structure and process indicators can help us evaluate whether our antibiotic stewardship efforts are moving us in the right direction [13–16]. A frequently asked question is whether behavioural change interventions lead to more appropriate antibiotic use, often measured as adherence to guidelines or profile of antibiotic consumption [3, 6, 17]. An equally important question is whether appropriate antibiotic use leads to the desired outcomes, like reduction in bacterial resistance rates, adverse events and mortality [3, 18]. Overprescribing outside guidelines often result from fear for the patients’ wellbeing, and are linked to patients who are severely ill or have an unclear diagnosis [19]. The expectation of clinicians’ to change their antibiotic prescribing behaviours needs to be supported by evidence-based guidelines and expert advice to reassure clinicians that guideline adherent antibiotic prescribing is safe and effective.

Readmission as an outcome measure in relation to antibiotic prescribing is not frequently reported [18]. Three studies within community acquired pneumonia show no association between guideline adherence and 30-day readmission, which is in agreement with the findings in this present study [20–22].

Evidence on the association between guideline adherence and mortality is diverse. Arnold et al. found that in-hospital mortality in patients receiving guideline-adherent treatment for community acquired pneumonia was 8% (95% CI, 7–10%), compared to 17% (95% CI, 14–20%) in the group of nonadherence [23]. Asadi et al. did not find any effect on mortality alone when looking at this variable in hospitalised patients with community acquired pneumonia, although the composite endpoint of death or ICU-admissions favoured guideline adherence [24]. In a Danish study of CAP, with similar resistance rates and treatment guidelines as Norway, Egelund et al. found that patients treated with guideline adherent penicillin monotherapy had lower CURB-65 score, less comorbidity and less in-hospital mortality in unadjusted analysis, while no association between mortality and guideline adherence was found in adjusted analysis [25]. However, a systematic review by Schuts et al., including 37 studies, showed that when empirical therapy was prescribed according to guidelines, the relative risk reduction of mortality was 35% [18]. The majority of patients included in these studies had pulmonary infections. These patients constituted almost half of our patient material. Our findings are coherent with this recent review, as we found that the odds ratio of in-hospital and 30-day mortality for the entire patient material was 0.46 and 0.48, respectively when guidelines were followed.

LOS was also favourably associated with adherent treatment in this study. The SHR was 1.17 for patients with guideline-adherent treatment, meaning that the rate of discharge was 17% higher for this group compared to the rate for the non-adherent group. Although not significant, there was a trend towards shorter LOS when analysed with linear regression analysis. 0.47 days constitutes 6.8% of the mean LOS for the study population (6.9 days) and 10.9% of a mean hospital stay in Norway, which is currently 4.3 days for patients outside the psychiatric wards [26]. The finding is in line with Schuts et al. which found that LOS was lower in 17 of the 24 included studies assessing association between adherence to guideline and LOS, favouring adherence [18]. The studies included in this review did however mainly include patients with lower respiratory tract infections, while our cohort had a large diversity of infectious diseases, and a maximum LOS of 21 days.

In observational cohort studies, the major limitation will be the potential for selection bias, in this case meaning that the patients with less severe illness may be more likely to receive guideline-adherent treatment [3]. By adjusting for indication for treatment, comorbidity, age, sex and seasonality, we have aimed to reduce the chance of confounding, but there could be differences in severity within each of the groups of indications, which could explain some of the difference seen in mortality between the adherent and non-adherent group. We did not have data on severity score, which could have helped us limit this factor. The grouping of indications is both a strength and a limitation. Looking at patient outcome and adherence across some of the most common infections seen in hospitals, makes the results more generalizable, but may also be more difficult to interpret. When working diagnosis on initiation of treatment was uncertain (eg “suspected UTI”) or there were more than one working diagnoses, we assessed adherence based on the most likely indication for treatment. Using working diagnoses for this purpose is limiting the generalizability of the results to individual groups of patients with more strict definitions of diagnoses. It does however reflect the daily challenge in the clinical setting where decisions about treatment have to be made before all diagnostic tools have been applied and results received and indicates that adhering to the most relevant guideline is a strength in this situation.

There were more patients admitted from an institution in the non-adherent group. This may be because patients admitted from institutions have more co-morbid disease and therefore present with more challenging diagnoses. Physicians may also consider the risk of resistant pathogens as higher and therefore prescribe more broad-spectrum agents. Furthermore, patients admitted from other institutions may already have received first line agents. Patient characteristics such as age, sex and comorbidity were very similar between the groups of patients receiving adherent or non-adherent treatment according to guidelines. The groups of UTI’s, “other” infections and GI-infections were however larger in the non-adherent group and LRTIs were larger in the adherent group. Prescribing for pneumonia and COPD exacerbations was the focus of the audit with feedback performed in the study wards in the underlying intervention study [6]. The mix of patients within the groups of indications varied to some extent, such as a higher number of pyelonephritis in the non-adherent group (38.8%) compared to the adherent group (23.2%) and higher number of sepsis with abdominal focus in non-adherent group (4.5%) compared to adherent group (0.6%). In a sensitivity analysis for 30-day mortality, the grouped indications were substituted with the individual indications. This only changed the estimated OR slightly, to 0.53. The difference seen between the groups can therefore not be explained by these factors alone. Another mechanism is of course that treatment recommended in guidelines is best practice - securing evidence based effective treatment of the infection, while minimizing ecologic effects, side effects and impact on the microbiotia and therefore is associated with better patient outcomes than non-adherent treatment.

We analysed according to the first indication for treatment, which was usually a working diagnosis on admission to the hospital. The diagnosis may have changed during the hospital stay. We therefore did a sensitivity analysis for 30-day mortality, where indication for treatment was substituted with discharge diagnosis. The OR for the association between adherent treatment and mortality only changed slightly, from 0.48 to 0.51.

Thirty-day readmission was defined as readmissions to the same hospital that the patient was discharged from. This could have caused an underestimation of readmissions if the patients were readmitted to other hospitals. As inclusion of patients were limited to a LOS of a maximum of 21 days, the mean LOS may be underestimated. Adherence to guideline within the group of excluded patients was not collected and is therefore unknown.

This was a multicentre study with patients included from three hospitals and three specialties, which increases generalizability. The number of included patients is also substantial and we adjusted for known risk factors for morbidity and mortality, such as age, comorbidity and admittance from an institution. Given that patients with a LOS longer than 21 days were excluded, this limits generalizability of the estimate for this outcome.

Norwegian guidelines were developed with broad involvement of more than 80 clinicians from all over the country [5, 27]. They are prudent, with mainly narrow-spectrum antibiotics as first-line empirical treatment [5]. It is of great importance that guidelines constitute best practice, to provide security for both the patient and the treating clinician, and secures standardized, safe and effective antibiotic treatment, also in the absence of an infectious diseases specialist.

This study builds on findings in previous studies, indicating that up-to-date, hospital antibiotic guidelines are safe and are associated with favourable clinical outcomes for inpatients. Antibiotic guidelines should be developed and regularly updated to ensure that they always promote best practice in the treatment of infectious diseases in the local context. Accurate, structured and easy-to-access documentation on severity of infections should be included in the electronic medical record to secure availability of this data in quality improvement processes, evaluation of treatment and research.

To be able to control for more factors in analyses, future studies should aim to collect information about severity of infections and whether empirical treatment provided adequate coverage for the individual patients.

## Conclusion

Empirical treatment according to guidelines on initiation of antibiotic therapy is associated with favourable clinical outcomes, such as in-hospital and 30-day mortality in our population of hospital inpatients.

## Additional file

Additional file 1: **Table S1.** Length of stay analysed with competing risk analysis (Fine and Gray). (DOCX 19 kb)

## Footnotes

1 CRB-65 (Severity assessment for pneumonia: Confusion, raised Respiratory rate, low Blood pressure and age 65 years or more).

## References

[1] Dyar OJ, Huttner B, Schouten J, Pulcini C (2017). What is antimicrobial stewardship?. Clin Microbiol Infect.

[2] Pulcini C., Binda F., Lamkang A.S., Trett A., Charani E., Goff D.A., Harbarth S., Hinrichsen S.L., Levy-Hara G., Mendelson M., Nathwani D., Gunturu R., Singh S., Srinivasan A., Thamlikitkul V., Thursky K., Vlieghe E., Wertheim H., Zeng M., Gandra S., Laxminarayan R. (2019). Developing core elements and checklist items for global hospital antimicrobial stewardship programmes: a consensus approach. Clinical Microbiology and Infection.

[3] Hulscher M, Prins JM (2017). Antibiotic stewardship: does it work in hospital practice? A review of the evidence base. Clin Microbiol Infect.

[4] NORM/NORM-VET. Usage of Antimicrobial Agents and Occurrence of Antimicrobial Resistance in Norway 2017, vol. 2018. Tromso/Oslo: Norwegian Surveillance System for Antibiotic Resistance in Microbes (NORM), Norwegian Veterinary Institute, Norwegian Institute of Public Health. https://unn.no/Documents/Kompetansetjenester,%20-sentre%20og%20fagråd/NORM%20-%20Norsk%20overvåkingssystem%20for%20antibiotikaresistens%20hos%20mikrober/Rapporter/NORM_NORM-VET_2017.pdf. Accessed 26 Sep 2018

[5] Norwegian Directorate of Health (2013). Norwegian National Clinical Guideline for antibiotic use in hospitals.

[6] Wathne JS, Kleppe LKS, Harthug S, Blix HS, Nilsen RM, Charani E (2018). The effect of antibiotic stewardship interventions with stakeholder involvement in hospital settings: a multicentre, cluster randomized controlled intervention study. Antimicrob Resist Infect Control.

[7] The Norwegian Tax Administration. The Norwegian National Registry. 2018. https://www.skatteetaten.no/en/person/national-registry/. Accessed 14 Sep 2018.

[8] WHO Collaborating Centre for Drug Statistics Methodology (2017). ATC index with DDDs. Norwegian Insitute of public health.

[9] Charlson ME, Pompei P, Ales KL, MacKenzie CR (1987). A new method of classifying prognostic comorbidity in longitudinal studies: development and validation. J Chronic Dis.

[10] Quan H, Li B, Couris CM, Fushimi K, Graham P, Hider P (2011). Updating and validating the Charlson comorbidity index and score for risk adjustment in hospital discharge abstracts using data from 6 countries. Am J Epidemiol.

[11] Stagg V. Charlson: Stata module to calculate Charlson index of comorbidity. Hosted by Orebro University School of Business, Sweden. Boston: Boston College Department of Economics; 2017. https://econpapers.repec.org/software/bocbocode/s456719.htm. Accessed 06 Dec 2018

[12] Austin PC, Fine JP (2017). Practical recommendations for reporting Fine-gray model analyses for competing risk data. Stat Med.

[13] Howard P, Huttner B, Beovic B, Beraud G, Kofteridis DP, Pano Pardo J (2017). ESGAP inventory of target indicators assessing antibiotic prescriptions: a cross-sectional survey. J Antimicrob Chemother.

[14] Berrevoets MA, Ten Oever J, Sprong T, van Hest RM, Groothuis I, van Heijl I (2017). Monitoring, documenting and reporting the quality of antibiotic use in the Netherlands: a pilot study to establish a national antimicrobial stewardship registry. BMC Infect Dis.

[15] Beović Bojana, Pulcini Céline, Dumartin Catherine, Béraud Guillaume, Nerat Barbara, Maurel Cristina, Doušak May, Čižman Milan, Allerberger Franz, Benko Ria, Berild Dag, Cunney Robert, Debacker Martine, Deptula Aleksander, Dumpis Uga, Dyar Oliver J, Ergonul Onder, Szabo Balint Gergely, Gormley Cairine, Grape Malin, Gudnason Thorolfur, Howard Philip, Huttner Benedikt, Ioannou Petros, Ionescu Ramona, Keuleyan Emma, Knepper Viviane, Kofteridis Diamantis, Kostyanev Tomislav, Krcmery Vladimir, Lakatos Botond, Luzzati Roberto, ten Oever Jaap, Pagani Leonardo, Pardo José Ramón Paño, Popescu Mihaela, Popovici Mihaela, Paul Mical, Bix Hege Salvesen, Schouten Jeroen, Sneddon Jacqueline, Stevanović Goran, Wechsler-Fördös Agnes, de With Katja, Vlahović-Palčevski Vera, Zarb Peter (2018). Legal framework of antimicrobial stewardship in hospitals (LEASH): a European Society of Clinical Microbiology and Infectious Diseases (ESCMID) cross-sectional international survey. International Journal of Antimicrobial Agents.

[16] Pollack LA, Plachouras D, Sinkowitz-Cochran R, Gruhler H, Monnet DL, Weber JT (2016). A concise set of structure and process indicators to assess and compare antimicrobial stewardship programs among EU and US hospitals: results from a multinational expert panel. Infect Control Hosp Epidemiol.

[17] Davey P, Marwick CA, Scott CL, Charani E, McNeil K, Brown E, et al. Interventions to improve antibiotic prescribing practices for hospital inpatients. Cochrane Database Syst Rev. 2017. 10.1002/14651858.CD003543.pub4:(2). 10.1002/14651858.CD003543.pub4 PMC6464541 28178770

[18] Schuts EC, Hulscher MEJL, Mouton JW, Verduin CM, Stuart JWTC, Overdiek HWPM (2016). Current evidence on hospital antimicrobial stewardship objectives: a systematic review and meta-analysis. Lancet Infect Dis.

[19] Skodvin B, Aase K, Charani E, Holmes A, Smith I (2015). An antimicrobial stewardship program initiative: a qualitative study on prescribing practices among hospital doctors. Antimicrob Resist Infect Control.

[20] Menendez R, Reyes S, Martinez R, de la Cuadra P, Manuel Valles J, Vallterra J (2007). Economic evaluation of adherence to treatment guidelines in nonintensive care pneumonia. Eur Respir J.

[21] Reyes Calzada S, Martínez Tomas R, Cremades Romero MJ, Martínez Moragón E, Soler Cataluña JJ, Menéndez Villanueva R (2007). Empiric treatment in hospitalized community-acquired pneumonia. Impact on mortality, length of stay and re-admission. Respir Med.

[22] Fanning M, McKean M, Seymour K, Pillans P, Scott I (2014). Adherence to guideline-based antibiotic treatment for acute exacerbations of chronic obstructive pulmonary disease in an Australian tertiary hospital. Intern Med J.

[23] Arnold FW, LaJoie A, Brock GN (2009). Improving outcomes in elderly patients with community-acquired pneumonia by adhering to national guidelines: community-acquired pneumonia organization international cohort study results. Arch Intern Med.

[24] Asadi L, Eurich DT, Gamble JM, Minhas-Sandhu JK, Marrie TJ, Majumdar SR (2013). Impact of guideline-concordant antibiotics and macrolide/beta-lactam combinations in 3203 patients hospitalized with pneumonia: prospective cohort study. Clin Microbiol Infect.

[25] Egelund GB, Jensen AV, Andersen SB, Petersen PT, Lindhardt BØ, von Plessen C (2017). Penicillin treatment for patients with community-acquired pneumonia in Denmark: a retrospective cohort study. BMC Pulm Med.

[26] Norwegian Directorate of Health. Activity data for Norwegian specialist health services 2017 (Aktivitetsdata for somatisk spesialisthelsetjeneste). 2018. https://helsedirektoratet.no/Lists/Publikasjoner/Attachments/1435/Somatikk_Årsrapport_2017.pdf. Accessed 27 Sept 2018.

[27] Feiring E, Walter AB (2017). Antimicrobial stewardship: a qualitative study of the development of national guidelines for antibiotic use in hospitals. BMC Health Serv Res.

[28] World Medical A (2013). World medical association declaration of Helsinki: ethical principles for medical research involving human subjects. JAMA..

