# Supplementary material for: The association between adherence to national antibiotic guidelines and mortality, readmission and length of stay in hospital inpatients: results from a Norwegian multicentre, observational cohort study
Source: Antimicrob Resist Infect Control. 2019 Apr 15;8:63. doi: 10.1186/s13756-019-0515-5 (PMC6466722; doi:10.1186/s13756-019-0515-5)
Supplement: Supplementary file 1 — Table S1. Length of stay analysed with competing risk analysis (Fine and Gray). (DOCX 19 kb) [file 13756_2019_515_MOESM1_ESM.docx]

**Supplement Table S1: Length of stay analysed with competing risk analysis (Fine and Gray)**

|  |  |  | **Length of stay (unadjusted)** | |  |  | **Length of stay (adjusted)** | |  |
| --- | --- | --- | --- | --- | --- | --- | --- | --- | --- |
|  | **(N=1756)** |  |  | **(N=1756)** |  |  |  | **(N=1756)** |  |
|  | **Median LOS**  **(Std.dev)** |  |  | **SHR (95%CI)** | **P** |  |  | **SHR (95% C.I.)** | **P** |
| **Adherence to guideline** |  |  |  |  |  |  |  |  |  |
| No | 6 (6,7) |  |  |  |  |  |  |  |  |
| Yes | 5 (5,6) |  |  | 1.20 (1.05, 1.36) | **0.006** |  |  | 1.17 (1.02, 1.34) | **0.025** |
|  |  |  |  |  |  |  |  |  |  |
| **Indication for**  **antibiotic treatment** |  |  |  |  |  |  |  |  |  |
| LRTI | 6 (6,6) |  |  | 1 |  |  |  | 1 |  |
| COPD with LRTI | 6 (5,6) |  |  | 1.19 (1.09, 1.30) | **<0.001** |  |  | 1.24 (1.09, 1.42) | **0.001** |
| Sepsis | 6 (5,7) |  |  | 1.05 (0.93, 1.19) | 0.431 |  |  | 0.99 (0.89, 1.11) | 0.915 |
| SSTI | 5 (4,5) |  |  | 1.44 (1.20, 1.72) | **<0.001** |  |  | 1.17 (0.96 1.42) | 0.115 |
| GI-infection | 7 (6,8) |  |  | 1.02 (0.83, 1.26) | 0.820 |  |  | 0.91 (0.74, 1.11) | 0.349 |
| UTI | 6 (5,6) |  |  | 1.17 (1.05, 1.31) | **0.004** |  |  | 1.13 (0.98, 1.30) | 0.102 |
| Other infections | 6 (5,7) |  |  | 1.03 (0.88, 1.21) | 0.690 |  |  | 1.03 (0.87, 1.21) | 0.767 |
|  |  |  |  |  |  |  |  |  |  |
| **Charlson**  **Comorbidity Index** |  |  |  |  |  |  |  |  |  |
| CCI = 0 | 5 (5,5) |  |  | 1 |  |  |  | 1 |  |
| CCI = 1 | 6 (5,6) |  |  | 0.83 (0.68, 1.02) | 0.080 |  |  | 0.88 (0.72, 1.07) | 0.201 |
| CCI = 2 | 7 (6,7) |  |  | 0.70 (0.64, 0.77) | **<0.001** |  |  | 0.78 (0.67, 0.91) | **0.002** |
| CCI = 3 | 6 (5,7) |  |  | 0.68 (0.52, 0.87) | **0.003** |  |  | 0.74 (0.55, 1.00) | 0.055 |
| CCI = 4 | 7 (6,12) |  |  | 0.47 (0.28, 0.78) | **0.003** |  |  | 0.52 (0.31, 0.89) | **0.016** |
| CCI > 4 | 8 (7,11) |  |  | 0.43 (0.41, 0.47) | **<0.001** |  |  | 0.50 (0.46, 0.55) | **<0.001** |
|  |  |  |  |  |  |  |  |  |  |
| **Age** |  |  |  |  |  |  |  |  |  |
| <=45 | 4 (4,5) |  |  | 1 |  |  |  | 1 |  |
| 46-65 | 6 (5,6) |  |  | 0.78 (0.74, 0.83) | **<0.001** |  |  | 0.84 (0.79, 0.90) | **<0.001** |
| 66-85 | 6 (6,7) |  |  | 0.63 (0.57, 0.69) | **<0.001** |  |  | 0.71 (0.64, 0.76) | **<0.001** |
| >85 | 6 (6,7) |  |  | 0.57 (0.43, 0.76) | **<0.001** |  |  | 0.68 (0.50, 0.93) | **0.015** |
|  |  |  |  |  |  |  |  |  |  |
| **Sex** |  |  |  |  |  |  |  |  |  |
| Male | 6 (6,6) |  |  | 1 |  |  |  | 1 |  |
| Female | 6 (5,6) |  |  | 1.11 (1.04, 1.19) | **0.002** |  |  | 1.09 (0.99, 1.20) | 0.065 |
|  |  |  |  |  |  |  |  |  |  |
| **Admitted from**  **institution** |  |  |  |  |  |  |  |  |  |
| No | 6 (5,6) |  |  | 1 |  |  |  | 1 |  |
| Yes | 6 (6,7) |  |  | 0.78 (0.68, 0.89) | **<0.001** |  |  | 0.85 (0.75, 0.96) | **0.011** |
|  |  |  |  |  |  |  |  |  |  |
| **Week of admission*** |  |  |  | 1.02 (1.01, 1.02) | **<0.001** |  |  | 1.01 (1.00, 1.02) | **0.008** |

*Adjustment for seasonality was performed by using the week of admission as adjustment variable.

All variables are included in adjusted analysis. Length of stay was analysed using competing risk-regression with in-hospital mortality as competing risk and with adjustment for clustering. P-values in boldface are statistically significant (p < 0.05).
